# Structure and Stability Characterization of Natural Lake Pigments Made from Plant Extracts and Their Potential Application in Polymer Composites for Packaging Materials

**DOI:** 10.3390/ma15134608

**Published:** 2022-06-30

**Authors:** Bolesław Szadkowski, Małgorzata Kuśmierek, Magdalena Śliwka-Kaszyńska, Anna Marzec

**Affiliations:** 1 Institute of Polymer and Dye Technology, Faculty of Chemistry, Lodz University of Technology, Stefanowskiego 16, 90-537 Lodz, Poland; malgorzata.kusmierek@dokt.p.lodz.pl; 2 Department of Organic Chemistry, Faculty of Chemistry, Gdansk University of Technology, Narutowicza 11/12, 80-233 Gdansk, Poland; magkaszy@pg.edu.pl

**Keywords:** plant extracts, lake pigments, pigment stability, UV aging, polymer coloration

## Abstract

Natural dyes were extracted from various plant sources and converted into lake pigments based on aluminum and tin. Three different plants (weld, Persian berries, and Brazilwood) were chosen as representative sources of natural dyes. High-performance liquid chromatography (HPLC) and triple-quadrupole mass spectrometry (QqQ MS) were used to identify dyestuffs in the raw extracts. The natural dyes and lake pigments were further characterized by optical and scanning electron microscopy (SEM), UV-Vis spectrophotometry, and thermogravimetric analysis (TGA). The stabilization of the studied plant extracts onto aluminum and tin salts led to the formation of natural lake pigments characterized by different color shades. The natural lake pigments showed improved thermal and chemical stability, which was confirmed by their higher degradation temperatures and lower solubility in chemical agents compared to natural dyes extracted from plants. This improvement can be attributed to electrostatic attraction due to the process of chelation. Ethylene-norbornene (EN) composites colored with the lake pigments exhibited uniform color and improved resistance to long-term UV exposure aging. After 300 h of UV exposure, the aging factor of the neat EN copolymer reduced to 0.3, indicating an advanced aging process of polymer compared to colored samples. Prolonged UV exposure deteriorated the mechanical properties of EN by approximately 57%, compared to about 43% with the application of BW/Al lake pigment. Natural lake pigments could be used as effective substitutes for commercial colorants in plastics for packaging applications.

## 1. Introduction

Dyes or pigments are used as colorants for polymers. Dyes are organic compounds that are soluble in, or have an affinity for, the medium being colored [1]. Pigments are chemical substances characterized by insolubility and the ability to create stable fine-grained dispersions with high tinctorial strength in most mediums. The most prominent uses of pigments are in paints and varnishes. They are also used extensively in the printing, paper, and polymer industries [2]. Depending on their chemical structures, pigments can be classified as organic, inorganic, or organic–inorganic colorants. Organic–inorganic colorants combine the best properties of organic pigments (such as a broad range of colors and colors with high intensity) with the advantages of inorganic pigments (such as high resistance to solvents, light, and temperature) [3,4]. The inorganic part provides dye stabilization and can simultaneously improve the functional properties of polymer composites (such as their thermal stability or barrier properties) [5,6,7,8,9].

Studies have shown that acidic dyes can be stabilized by adsorption on TiO_2_ and silica surfaces previously modified with aminosilanes [10,11]. Coloring agents may also be obtained by the adsorption of carminic acid on a cation exchange layer of filler–montmorillonite [12]. An interesting trend is the preparation of organic–inorganic pigments using layered hydroxides [13,14,15,16]. The application of layered hydroxides modified with dye can markedly improve the barrier stability and flame retardancy of polymer composites [17]. Conventionally, organic–inorganic pigments are obtained by the laking process. Laking involves the precipitation of water-soluble dyes or extracts with different salts, such as alum, tin chloride, or copper sulfate [18]. These salts can form complexes with the dye molecules via coordinate bonds. Different metal salts can be used as mordants in the process. Inorganic substrates (e.g., aluminum hydroxides) may also be used, producing lakes known as toners [18,19]. These complexes are insoluble and hence improve the staining ability of a dye as well as its fastness. Therefore, a water-soluble dye can be converted into an insoluble pigment by making it into a lake. Due to the simple process of production and the superior properties of lakes, they make promising colorants and have attracted the interest of researchers and industry. In the last century, natural pigments were replaced by synthetic colorants with higher photo-stability, thermal and chemical stability, and low prices, as well as a wider variety of colors. However, increasing ecological awareness and the poisonous properties of some synthetic colorants have renewed interest in natural dyes and pigments, which are characterized by low toxicity and biodegradability [19].

In recent years, many researchers have focused on developing new natural lake pigments with improved chemical properties and high stability for possible applications in polymers used as packaging film. Deveoglu et al. [20] successfully prepared lake pigments from hemp (*Datisca cannabina* L.) using KAl(SO_4_)_2_·12H_2_O (alum), FeSO_4_·7H_2_O, and SnCl_2_·2H_2_O mordants. Zhou et al. [21] used Chinese Tallow leaf extract and different mordants (two metal and one biomordant) to obtain wool fabric dye. The dye exhibited excellent antioxidant activity, good UV protection, and high antibacterial activity against *Escherichia coli *(*E. coli*)** and *Staphylococcus aureus *(*S. aureus*)**. Mahmud-Ali et al. [22] applied aluminum salts Al_2_(SO_4_)_3_·14–15H_2_O and KAL (SO_4_)_2_·12H_2_O to precipitate dyes from aqueous extracts of onion peel and Canadian Goldenrod. Many other studies have contributed to a better understanding of the conditions of lake pigment formation [23,24].

Many fillers have been investigated as potential carriers for lake pigments. For instance, Sirirak et al. [25] prepared lake pigments based on *Caesalpinia Sappan* Linn. and aluminum hydroxide using the adsorption method. The prepared red-pink lake pigments were utilized as a natural colorant in natural rubber (elastomer) toy balloons, which could make toy balloons safer for children.

Previously, we described the use of different fillers, including vermiculite, palygorskite, and sepiolite, to obtain organic–inorganic pigments by the laking method (precipitation) [26,27]. These obtained organic–inorganic pigments act as multifunctional additives in polymers, positively influencing their applicative performance, while simultaneously providing high aesthetic qualities. The present study is the first to investigate the behavior of lake pigments stabilized on salts in thermoplastic elastomers intended for use as packaging materials. Inspired by traditional recipes, the lake pigments from weld (*Reseda luteola* L.) (W), Persian berries (*Rhamnimaturi*) (PB), and Brazilwood (*Caesalpinia Sappan* Linn.) (BW) extracts were obtained. The usefulness of the lake pigments as natural colorants in the thermoplastic elastomer was investigated using different techniques. The obtained pigments could potentially be applied as colorants in natural composites or polymers for food packaging.

## 2. Materials and Methods

### 2.1. Reagents and Raw Materials

Acetonitrile and methanol of HPLC grade were purchased from Merck (Darmstadt, Germany). Hydrofluoric acid (48% in water), SnCl_2_, and KAl(SO_4_)_2_ × 12 H_2_O were purchased from Sigma-Aldrich (Steinheim, Germany). All aqueous solutions were prepared using deionized Milli Q water. Weld (*Reseda luteola* L.), Persian berries (*Rhamnimaturi*), and Brazilwood (*Caesalpinia Sappan* Linn) were obtained from Kremer Pigmente (Aichstetten, Germany) in dried form. Ethylene-norbornene random copolymer, with the commercial name Topas Elastomer E140 (EN), was provided by TOPAS Advanced Polymers (Raunheim, Germany).

### 2.2. Extraction of Dyes from Weld, Persian Berries, and Brazilwood

The plant materials (ca. 10 g) were homogenized and then suspended in 500 mL of aqueous methanol mixture (3:1 *v*/*v*) containing 10 mL of 4 M aqueous hydrofluoric acid (HF) solution. The mixtures were refluxed for 1 h, cooled to 50 °C, and filtered under reduced pressure. Next, 1 mL of each supernatant was taken up in 2 mL ACN/MeOH/DMSO (1:1:1, *v*/*v*), of which 2 μL was injected into an HPLC column.

### 2.3. Lake Pigment Preparation Method

#### 2.3.1. Lake Pigments from Weld, Persian Berries, and Brazilwood with Alum (RE/Al, JP/Al, BW/Al)

To the hot extracts of weld, Persian berries, and Brazilwood, 20 g of KAl(SO_4_)_2_ × 12 H_2_O was added. The mixtures were heated for 30 min at a temperature not exceeding 60 °C. After cooling, 30 g of CaCO_3_ was added in portions (foaming). The suspensions were left for 24 h for precipitation. The pigments were filtered, washed, and air-dried.

#### 2.3.2. Lake Pigment from Weld with Tin (RE/Sn)

To the hot extract of weld, 20 g of SnCl_2_ was added. The mixture was refluxed for 30 min, cooled, and left for 24 h. The solid precipitate was filtered, washed, and air-dried.

### 2.4. Preparation of Polymer Composites

Four lake pigments were used as new colorants for ethylene-norbornene copolymer materials. Colorful EN composites were prepared using 100 phr (parts per hundred parts of rubber) of EN copolymer mixed with 3 phr of lake pigment. Mixing was performed in an internal micromixer equipped with cylindrical blades (Brabender, Duisburg, Germany) with the following process parameters: chamber temperature 110 °C and rotor speed 50 rpm. Composite plates were prepared on a hydraulic compression press configured to 120 °C with 15 MPa pressure for 10 min.

### 2.5. Characterization Techniques

#### 2.5.1. Chromatographic Analysis

Chromatographic analyses were performed using the Agilent 1290 system (Agilent technology, Waldbronn, Germany) controlled by Agilent Masshunter software version B 06.01. The parameters for the spectrochromatographic analysis are presented in Table 1.

#### 2.5.2. Thermogravimetric Analysis

The thermal characteristics of the natural lake pigments were determined using a TGA thermogravimetric analyzer (Mettler Toledo, Columbus, OH, USA). Powder samples of approximately 10 mg were placed in an alumina crucible and heated from 25 °C to 600 °C under an argon atmosphere at a heating rate of 10 °C/min. Thermogravimetric analysis was performed in triplicate runs for each sample.

#### 2.5.3. Chemical Resistance Characteristic

The chemical resistance of each of the natural dyes and prepared lake pigments was evaluated by immersing the samples (~10 mg) in two solvents with different polarities: ethanol and hexane. Subsequently, the colored supernatants were analyzed by UV-Vis spectrophotometry in the range 200–1100 nm using a quartz cuvette.

#### 2.5.4. UV-Vis Spectroscopy

The UV-Vis spectra of the lake pigments were recorded on an Evolution 201/220 UV-Visible spectrophotometer (Thermo Scientific, Waltham, MA, USA). Prior to the measurements, the samples were stored under normal conditions and spectra were recorded in the range 200–1100 nm, directly in the air without further pre-treatment.

#### 2.5.5. Optical Microscopy

Optical microscopy images of the lake powders and polymer composites were collected using an Optatech optical microscope (Optatech, Warsaw, Poland) operating at 64× or 100× magnification, coupled with a Leica MZ 6 camera (Leica microsystems, Wetzlar, Germany) and OptaView software (Optatech, Warsaw, Poland).

#### 2.5.6. Scanning Electron Microscopy (SEM)

Scanning electron microscopy (SEM) was used to investigate the morphology of the natural lake pigments powders. SEM images with the magnification of 25 k× were collected using a scanning electron microscope (SEM, Zeiss, ULTRA Plus, Oberchoken, Germany), operating at an accelerating voltage of 15 keV. The samples were coated with carbon to avoid a charging effect.

#### 2.5.7. Colorimetric Measurements

The color characteristics of the prepared EN composites colored with lake pigments were evaluated on a CM-3600d spectrophotometer (Konica Minolta Sensing, Osaka, Japan). The measurements were performed in the wavelength ranges of 360–740 nm, under standard illuminant D65 and 10° observer, and represented in CIELAB color space. The CIELAB results presented in the manuscript are the average values of the 10 measurements for each composite sample. Based on the colorimetric analysis, three different color coordinate parameters have been determined: L–brightness; a*–redness/greenness color coordinate; and b*–yellowness-blueness color coordinate.

#### 2.5.8. Carbonyl Index Determination (FTIR Measurements)

The infrared absorbance spectra were recorded using a Thermo Scientific Nicolet 6700 FTIR (Fourier transform infrared) spectrometer (Thermo Scientific, Waltham, MA, USA). The ATR-FTIR technique was used to examine the formation of oxidation products in the wavelength range of 4000–500 cm^−1^ (64 scans, resolution of 4 cm^−1^, absorption mode). Prior to the FTIR analysis, background measurements and baseline corrections were made. Changes in the relative absorbance intensity of the ketone group A_>C=O_ (corresponding to 1800–1680 cm^−1^) versus the absorbance intensity of the methylene group A_−CH_2_−_ (at 3000–2800 cm^−1^) were used to calculate carbonyl index (CI) according to Equation (1) [28]:

(1) $$\mathrm{CI}=\frac{A_{>C=O}}{A_{-{\mathrm{CH}}_{2}-}}$$

#### 2.5.9. Mechanical Properties of EN Composites

The mechanical properties of the EN/lake pigment composites were evaluated using a Zwick/Roell 1435 universal tensile testing machine at a uniform crosshead speed of 500 mm/min. The tests were carried out following the guidelines of the ISO 37 standard. The elongation at break (E_B_) and tensile strength (T_S_) were calculated as the average of five measurements. The measurement error determined for the mechanical parameters was approximately 10%.

#### 2.5.10. Accelerated UV Aging and Aging Factor Determination

Accelerated UV aging was performed in an Atlas UV 2000 apparatus. The procedure included two successively repeating segments: a day segment (irradiance 0.7 W/m^2^, temperature 60 °C, duration 8 h) and a night segment (no UV radiation, temperature 50 °C, duration 4 h). The accelerated UV aging test was performed for 300 h using radiation at wavelength λ = 343 nm. The aging factor was determined based on tensile strength tests, where AF was evaluated according to Equation (2) [29]:

(2) $$\mathrm{AF}=\frac{{\left(T_{S}\cdot E_{B}\right)}_{\mathrm{after}\;\mathrm{aging}}}{{\left(T_{S}\cdot E_{B}\right)}_{\mathrm{before}\;\mathrm{aging}}}$$

## 3. Results and Discussion

### 3.1. Characterization of Plant Extracts by Liquid Chromatography-Mass Spectrometry

The colorants present in the plant materials were isolated using a mild extraction method with hydrofluoric acid. The use of hydrofluoric acid enables the isolation of natural dyes from various matrices (e.g., plant tissues, lake-pigments, oil paints, textiles) with high efficiency, without breaking the glycosidic bonds or causing other undesired transformations [30,31,32,33]. Liquid chromatography-mass spectrometry with atmospheric pressure negative electrospray ionization LC-ESI (−)-MS was applied to identify the main components in the plant extracts. The identification of colorants was performed based on retention times, UV, and mass spectra in the negative ionization mode (ESI(−)-MS). Mass spectra of flavonoids detected in weld and Persian berries showed the loss of small neutral fragments such as CO (−28 u), H_2_O (−18 u), and CO_2_ (−44 u), as well as of combinations of such fragments. Fragmentation reactions were observed for all the flavonoids analyzed in this study (Table 1). The detected fragment ions contributed to the mass spectral fingerprint. Another type of fragmentation of flavonoids is cleavage of the C-ring by a *retro*-Diels-Alder (RDA) mechanism. This leads to ^i,j^A^−^ and ^i,j^B^−^ ions, providing information on the number and type of substituents in the A^−^ and B^−^ rings [34,35]. The fragmentation pathway of *O*-glycosylated flavonoids starts with the cleavage of glycosidic bonds and elimination of the sugar moieties. The loss of 162 u (hexose) and 146 u (deoxyhexose) enables the determination of the carbohydrate sequence. In flavonoid *C*-glycosides, the major fragmentation pathways concern the cross-ring cleavages of the sugar residue. The LC-ESI(-)-MS analysis of the yellow extract of weld showed the presence of eleven flavone-type dyes. Most of them were present in *O*-glucoside form. In the extract of Persian berries, nine yellow flavone-glucosides and six aglycons were identified.

The fragmentation of coloring substances detected in Brazilwood (brazilin-type homoisoflavonoids) proceeded according to one of two mechanisms: the loss of small neutral molecules or the cleavage of internal rings [36]. The red extract of Brazilwood contained two main compounds, protosappanin B and brazilin, and a trace amount of brazilein.

**Figure 1:**
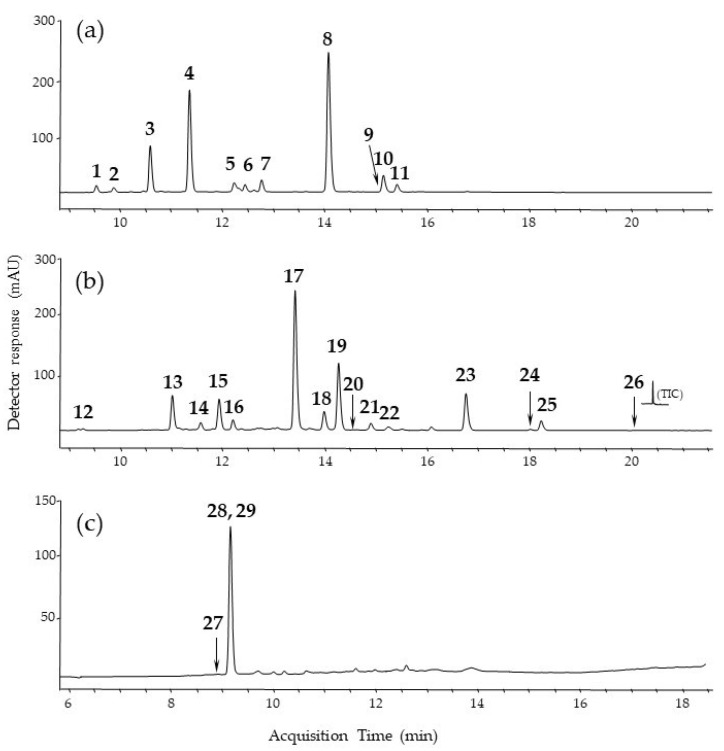
Chromatograms at 254 nm for extracts taken from (**a**) weld, (**b**) Persian berries, (**c**) Brazilwood.

The chromatographic profiles of the plant extracts are presented in Figure 1. The retention times, molecular ions, main fragment ions, elemental compositions, maximum absorbance wavelengths (λ_max_), and formulas for the detected dyes are summarized in Table 2. All of the extracts were used for the preparation of lake pigments by stabilization onto aluminum and tin salts.

### 3.2. Structural Characterization of Lake Pigments

**Figure 2:**
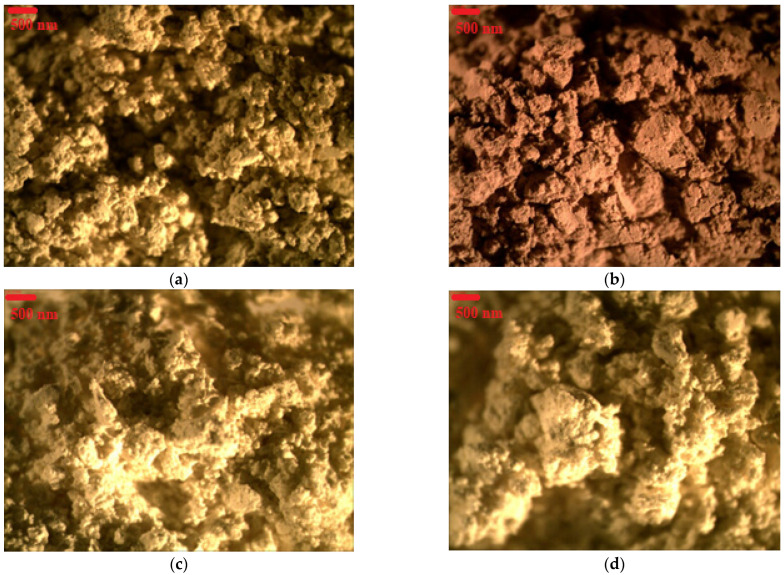
Microscopic images of natural lake pigments: (**a**) PB/Al; (**b**) BW/Al; (**c**) W/Al; (**d**) W/Sn.

Dyes derived from natural sources have attracted considerable attention in recent years, due to increasing ecological awareness. Many of the most common red, yellow, or blue natural colorants also exhibit broad biological activities. Flavonoids, for example, possess bioactive properties and are of great interest for nutrition and pharmacology, due to their extraordinary antioxidant, antibacterial, anti-inflammatory, antifungal, and antitumor properties [37,38,39]. Despite the many advantages of such colorants, they often do not exhibit adequate thermal, chemical, or light stability when applied in advanced materials. Therefore, three different natural plant extracts, weld (W), Persian berries (PB), and Brazilwood (BW) were selected to produce stable colorants with improved physico-chemical performance. The stabilization of these natural plant dyes on the aluminum and tin salts led to the formation of natural lake pigments characterized by different color shades. Figure 2 shows the optical microscopic images of the studied lake pigments. As can be seen, the PB/Al, W/Al and W/Sn pigments were light brown in color, while the BW/Al lake pigment was a deep brown color. The color differences were also reflected in the UV-Vis data shown in Section 2.4, in which characteristic UV-Vis absorption peaks can be distinguished at a wavelength of 415 nm for W/Al and at 550 nm for BW/Al. The stabilization of weld extract on tin resulted in a pigment of a slightly lighter color compared to the stabilization of weld on aluminum.

**Figure 3:**
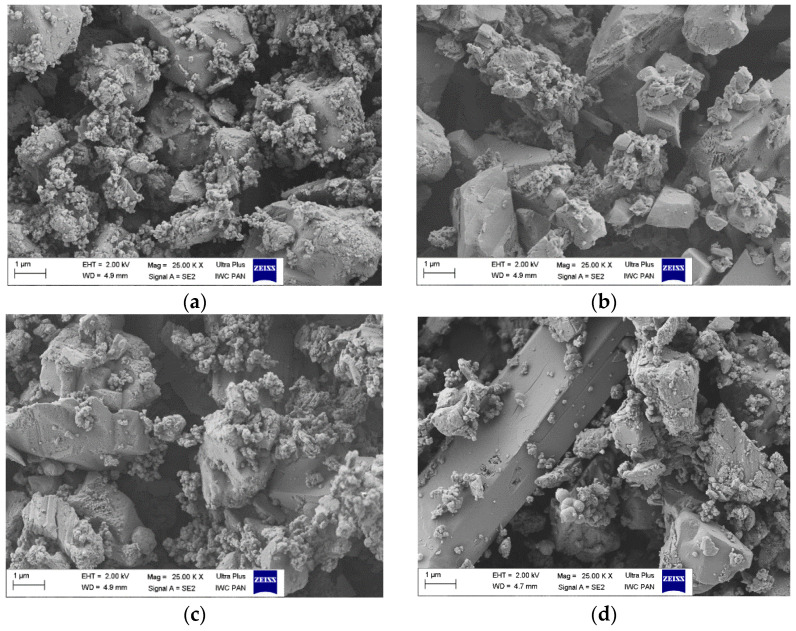
Scanning electron microscopic images of natural lake pigments: (**a**) PB/Al; (**b**) BW/Al; (**c**) W/Al; (**d**) W/Sn.

Figure 3 shows the SEM micrographs of the investigated powders. As can clearly be seen, the shapes and sizes of the particles of lake pigments varied depending on the inorganic matrix used. Regardless of the type of natural chromophore, the lake pigments made from aluminum hydroxide were characterized by a cubic morphology, with rough, uneven shapes and lateral dimensions of 1 × 2 μm, covered with many finer particles (Figure 3a–c). The tin-based lake pigment, depicted in Figure 3d, showed a similar shape to Al-based pigments, but with larger dimensions. The very long individual particles were a few microns in length with widths of approximately 2 μm.

### 3.3. Chemical Resistance of Lake Pigments

**Figure 4:**
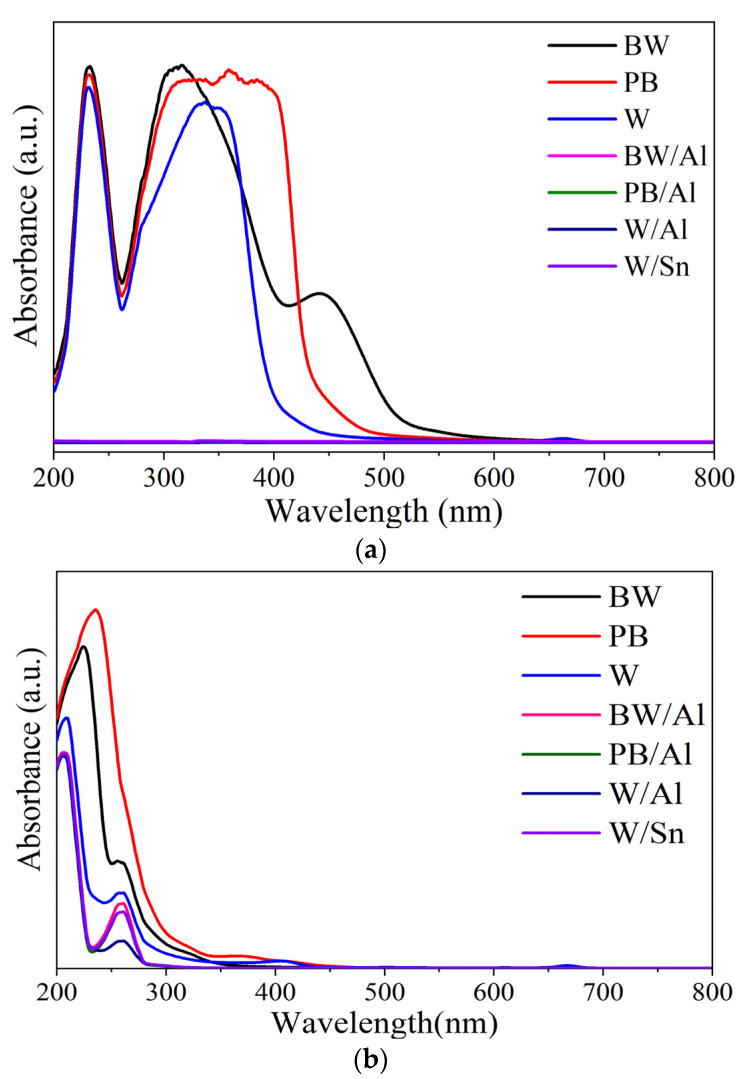
UV-Vis spectra of solutions of natural lake pigments with (**a**) ethanol and (**b**) n-hexane after 24 h of exposure.

Generally, the fabrication of lake pigments by complexing dyes with metal ions (often Al^3+^, Ca^2+^, Fe^3+^, or Sn^4+^) results in insolubility in the binding medium and produces colorants with various colors [40]. The next step was therefore to assess the effect of stabilization on the chemical resistance of the natural dyes. For this purpose, the samples of the natural dyes were immersed in two solvents with different polarities: ethanol and hexane. The colored solutions were analyzed by UV-Vis spectrophotometry. The obtained UV-Vis spectra are presented in Figure 4. The strong absorbance peaks on the UV-Vis spectra indicate the pronounced migration of dyes into the solvents. This is clearly evident in the case of ethanol solutions, for which intense characteristic UV-Vis peaks were noted at around 315 nm, 360 nm, and 340 nm for BW, PB, and W, respectively. In contrast to the natural dyes, the prepared lake pigments were found to be resistant to the selected solvents. This was confirmed by significantly lower absorption peaks registered by UV-Vis spectrophotometry measurements in the visible region. It can be assumed that the stabilization of the colorful plant extracts on Al or Sn resulted in colorants with higher resistance to chemical attacks. These results corroborate our previous studies, in which organic dyes showed improved chemical resistance to different solvents after stabilization on mineral supports [41,42].

### 3.4. Thermal Stability of Lake Pigments

**Figure 5:**
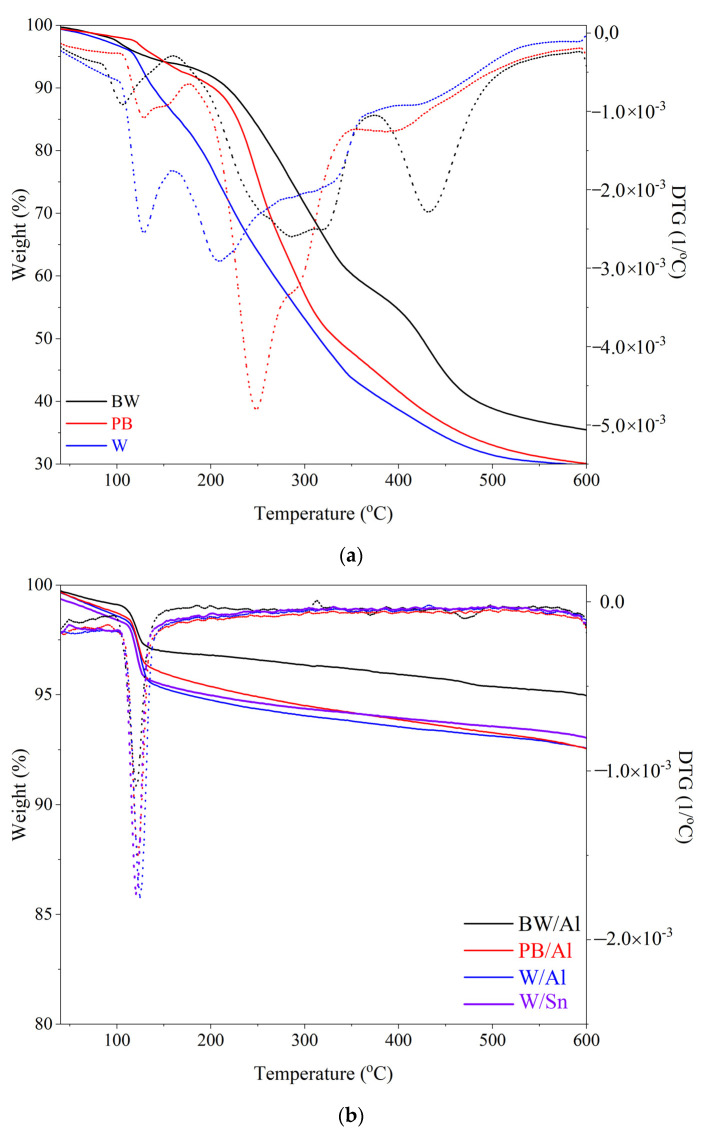
Thermogravimetric curves (TG/DTG) obtained for: natural dyes (**a**) and lake pigments (**b**).

The thermal properties of the natural dyes and Al- and Sn-based lake pigments were analyzed using thermogravimetric analysis (TGA). The TG/DTG profiles of the studied samples are presented in Figure 5. The corresponding degradation temperatures are presented in Table 3. Figure 5a shows the thermal decomposition of the natural colorants proceeding in several steps. The intensive process began at approximately 120–140 °C, with a total weight loss at 600 °C of around 30%. The poor thermal stability of the studied natural dyes is evidenced by their very low initial thermal decomposition temperatures (T_05_) at 118 °C, 130 °C, and 142 °C for W, BW, and PB, respectively. The TG/DTG results for the natural dyes are in agreement with the generally low thermal stability observed for most colorants derived from plant sources [43]. Importantly, the TG/DTG experiment revealed a shift of thermal degradation temperatures toward higher values for the designed natural lake pigments, indicating improved thermal stability after stabilization on the inorganic supports (Figure 5b). Moreover, high inorganic content was observed in all the natural lake pigment samples, which may be attributed to the complexation between Al and Sn metals and organic ligand species. Generally, the thermal stability of the studied Al-based lake pigments can be given in the following order: W/Al < PB/Al < BW/Al. The T_05_ temperature was higher for the W/Sn lake than for W/Al. By analogy to the thermal stability results reported for natural pigments prepared from *Datisca cannabina* L. [20], the strong metal-chelating capacity of the natural dyes present in plant extracts may be assumed to be responsible for the enhanced stability of the lake pigments.

**Figure 6:**
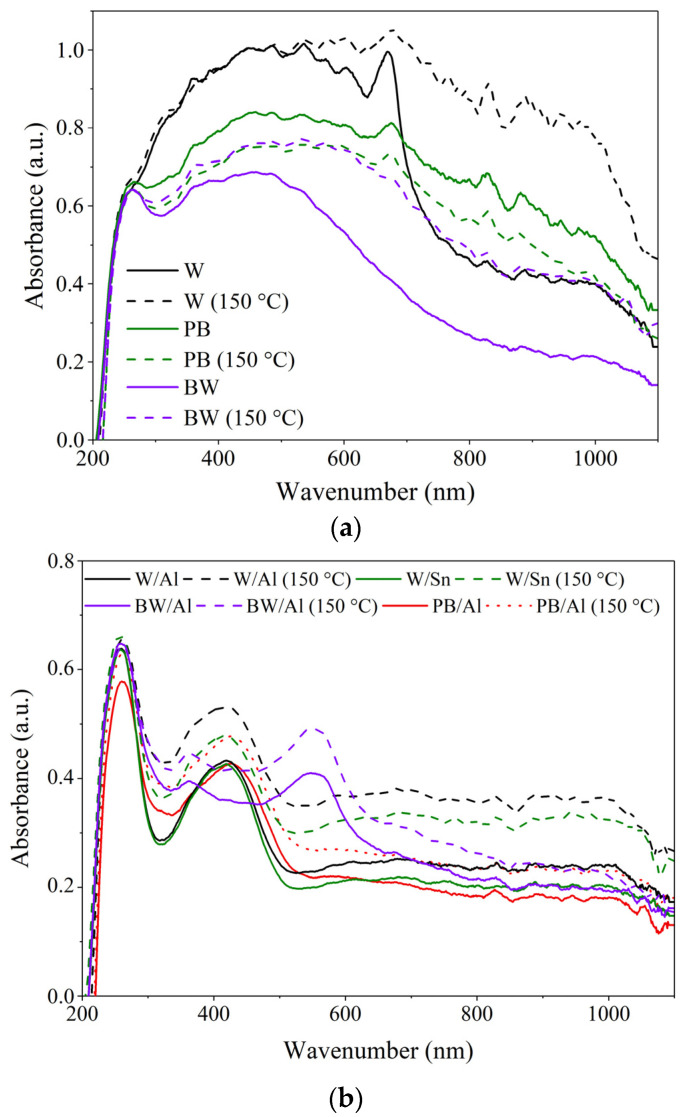
UV-Vis-NIR curves for: natural dyes (**a**,**b**) natural lake pigments exposed to elevated temperature of 150 °C.

The colorimetric thermal stability of each of the natural dyes and designed lake pigments was also evaluated based on UV-Vis spectrophotometric measurements after thermal treatment at 150 °C for 30 min. This procedure for evaluating color stability had been applied previously to different natural dyes and pigments [26,44]. The UV-Vis spectra of the studied samples are shown in Figure 6. The UV-Vis data for the natural dyes reveal significant changes in color following thermal treatment. As can be seen in Figure 6a, the UV-Vis spectra characteristics changed considerably, especially for W, confirming its very low thermal stability. The main characteristic UV-Vis peaks shifted from approximately 490 nm to 570 nm, from 480 nm to 540 nm, and from 460 nm to 550 nm for W, PB, and BW, respectively. In contrast, when the Al- and Sn-based lake pigments were exposed to 150 °C there was no shift of the characteristic UV-Vis peaks, which maintained their initial positions at 420 nm, 422 nm, 425 nm, and 555 nm for W/Sn, W/Al, PB/Al, and BW/Al, respectively. This means that the colors of the lake pigments remained stable across the tested temperature interval. It can be assumed that stabilization onto aluminum or tin improved the thermal stability of the colorants.

### 3.5. Application of Lake Pigments in Plastic

In the last stage of the research, the prepared natural lake pigments were incorporated at 3 wt% loading into ethylene-norbornene copolymer (EN) films to produce colorful plastic products. Ethylene-norbornene copolymer belongs to the group of cyclic olefin copolymers (COC) that are characterized by high purity, low permeability, and amorphous transparency [45,46]. Materials made from EN are used in medical, optics and food packaging materials, where color is often a crucial factor.

The EN composites were evaluated based on spectrophotometric measurements, according to the CIELAB color space model. The presence of natural lake pigments in the EN resulted in a remarkable variation in CIELAB coordinates (Table 4). Neat EN copolymer was characterized by a high lightness value (L = 86.52) due to its high transparency. The addition of lake pigments resulted in composites with lower L values, which can be attributed to intense selective light absorption and thus the effective hiding of the transparent neat copolymer. Moreover, b* values, expressing the yellow-blue component of the samples, shifted from 5.06 for the neat EN up to 9.57, 53.16, 40.96, and 38.09 for EN/BW/Al, EN/PB/Al, EN/W/Al, and EN/W/Sn, respectively. The a* values of the samples fell significantly compared to neat EN. These findings confirm that Al- and Sn-based lake pigments made from natural dyes are capable of providing polymer composites with very good color performance and suggest that the selection of the precipitated substrate may have a considerable impact on the final color of the material.

The prolonged utilization of polymer products leads to photo-degradation, which results in surface changes and leads to discoloration, making the final products aesthetically unappealing [47]. According to the literature, some natural additives can be effectively used to protect polymer composites against the negative effects of long-term light exposure [48,49]. For instance, Quiles-Carrillo et al. [50] report that the incorporation of natural gallic acid into HDPE leads to composites with significantly improved light stability, as shown by FTIR measurements. The most important factor causing the weathering of polyolefin composites is photo-oxidation, which occurs in the presence of oxygen and UV-radiation below 400 nm [51].

**Figure 7:**
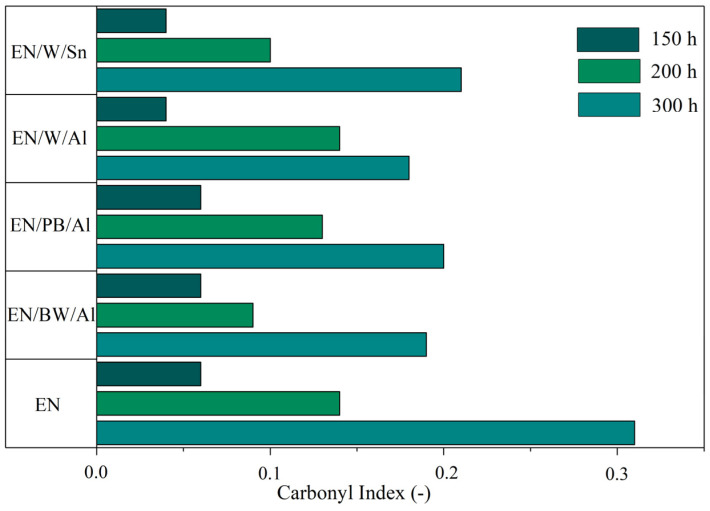
Carbonyl index determined for EN/natural lake pigment composites exposed to UV aging.

**Figure A1:**
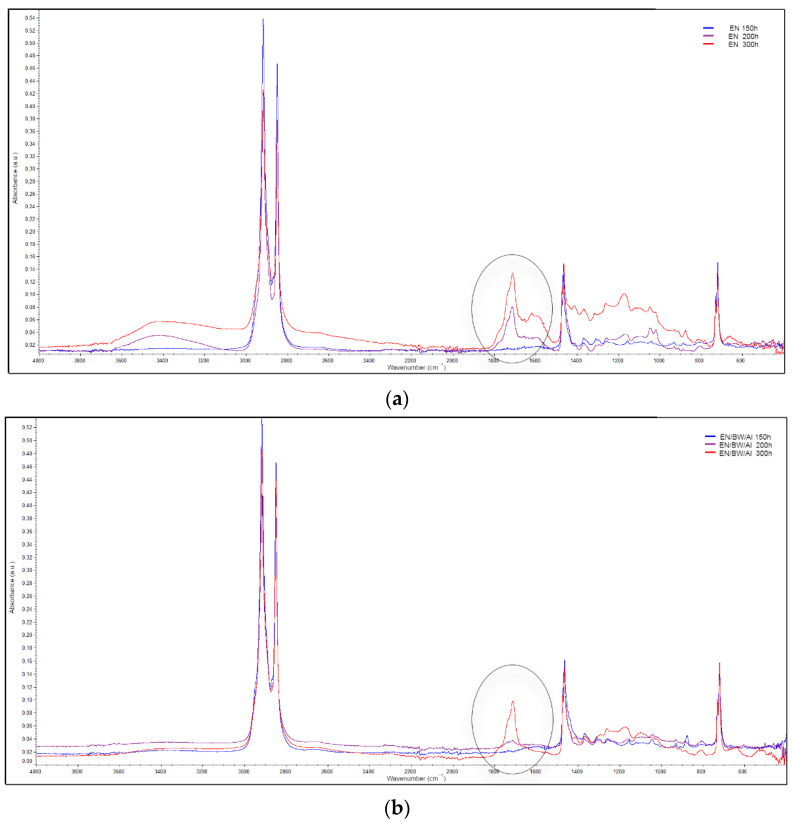

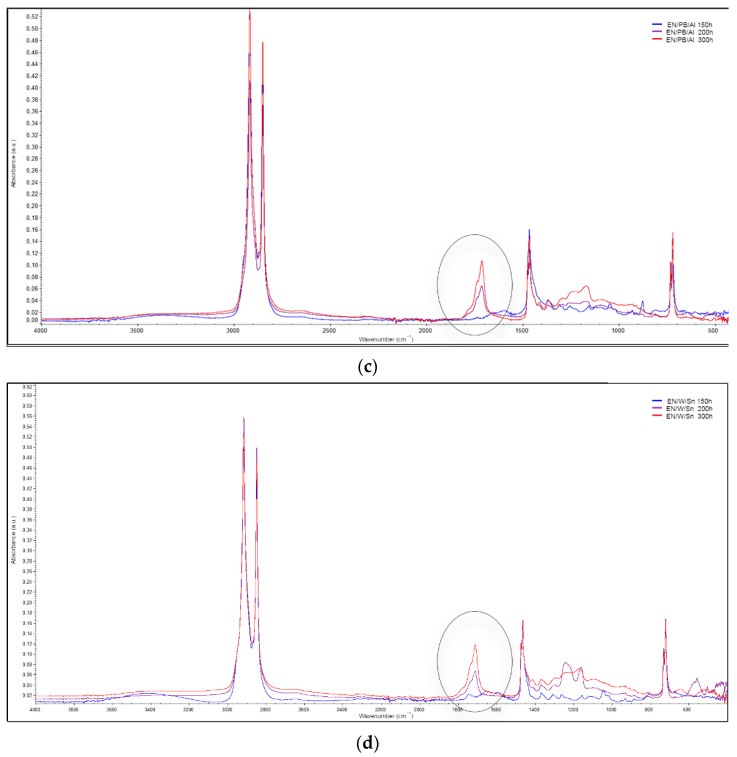
FTIR spectra of EN/lake pigments composites exposed to different time of UV radiation: (**a**) neat EN, (**b**) EN/BW/Al composites, (**c**) EN/PB/Al composites, (**d**) EN/W/Sn composites.

In this study, UV aging experiments on the colored EN composites for different exposure times were performed. The structural and physical changes were monitored based on FTIR measurements and mechanical tests. On the basis of the FTIR measurements of the aged composites, the intensity of carbonyl groups (C=O) at 1680–1800 cm^−1^ in the FTIR spectra was analyzed. The changes in the carbonyl index (CI) with increasing UV aging times are presented in Figure 7, while the FTIR spectra obtained for studied samples are presented in Appendix A (Figure A1).

As shown in Figure 7, UV aging time contributed to a progressive increase in the CI for all EN composites, reaching the highest values after 300 h of aging. Generally, a higher CI indicates a higher degree of polymer degradation. As expected, the uncolored EN copolymer showed a dramatic increase in C=O groups with UV exposure time, compared to the composites filled with lake pigments. After 300 h of aging, the CI for neat EN was almost double the CI of the other samples. This is reliable evidence of considerable photodegradation in the case of the EN copolymer upon UV exposure and simultaneously of the effective protection provided by natural lake pigments employed as colorants in this material.

**Figure 8:**
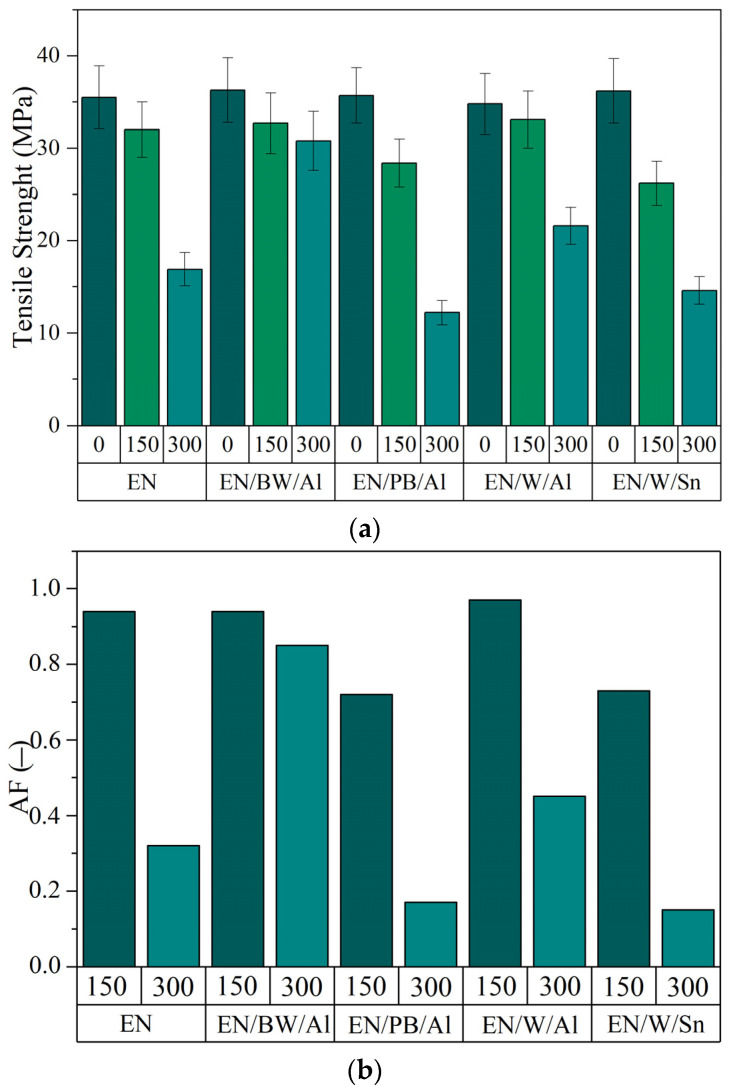
Mechanical parameters determined for EN/natural lake pigments composites exposed to UV aging: (**a**) tensile strength and (**b**) aging cofactor.

Photo-oxidation reactions are generally assumed to alter both the molecular weight and crystallinity of polymers, affecting their mechanical performance [52]. Therefore, tensile strength tests of the samples before and after UV aging were performed. The results are shown in Figure 8.

As shown in Figure 8, all EN samples displayed a similar progressive reduction in mechanical performance when subjected to UV aging. This is clearly evident for neat EN copolymer and EN/PB/Al, which showed strong reductions in tensile strength after 300 h of UV aging, from 36.0 MPa and 35.7 MPa up to 16.9 MPa and 12.2 MPa, respectively. The mechanical performance was also reflected in the aging factor values determined based on the changes in the tensile properties and elongation at break of the samples. As shown in Figure 8b, a similar decreasing trend was observed with increasing aging time. The lower aging factor values resulted from the reduction in tensile strength and elongation of the aged samples. This is consistent with the literature and is associated with oxidation-induced chain scission [53]. In comparison, the EN composites colored with BW/Al and W/Al lake pigments exhibited less pronounced reductions in tensile strength and aging factors following UV aging. These results are in good agreement with data obtained from FTIR measurements (CI) and with our previous research on other EN copolymer composites filled with natural pigments derived from plants [54]. The present findings prove that the selected natural lake pigments effectively protected EN material against photo-degradation due to their ability to absorb light radiation.

## 4. Conclusions

In this study, a multi-technical approach was used to investigate different natural lake pigments made from plant-derived natural dyes and their potential for use as new eco-friendly colorants for plastic packaging materials. The most important conclusions from this study are as follows:

▪ The lake pigments precipitated on tin and aluminum showed different color performance, depending on the natural dye and inorganic support.
▪ Importantly, the prepared lake pigments exhibited improved thermal and chemical stability compared to the raw natural dyes.
▪ The application of the studied lake pigments in ethylene-norbornene (EN) copolymer materials led to colorful products with improved resistance to long-term UV radiation.
▪ In most cases, the EN composites containing natural lake pigments exhibited lower reductions in their mechanical parameters after 300 h of UV exposure compared to the neat EN copolymer. The enhanced UV resistance of the colored composites was also confirmed by the lower content of C=O groups generated on their surfaces during aging.

Based on these results, natural lake pigments show great potential for use as promising candidates for eco-friendly colorants for plastics, exploiting renewable natural resources.

## Figures and Tables

**Table 1:** Conditions of chromatographic separation and detection of the examined dyes.

Column	Poroshell EC-C18 (3.0 × 150 mm), 2.7 μm, Agilent Technologies		
Column temperature	40 °C		
Injection volume	2 µL		
Flow rate	0.4 mL min^−1^		
Eluents	(A) 0.1 % HCOOH in water, (B) 0.1 % HCOOH in ACN/MeOH (1:1; *v*/*v*)		
Gradient program	Time, min	%A	%B
	0	90	10
	20	0	100
	30	0	100
UV–Vis detection	Wavelengths: 254, 350 nm		
ESI MS detection	Polarity negative		
Mode	Profile 50–1000 m/z Product ion 50–650 m/z		

**Table 2:** Spectrochromatographic data of components extracted from weld, Persian berries, and Brazilwood.

	Peak No.	t_R_ (min)	[M-H]^−^, (m/z)	Fragment Ions (m/z)	Elemental Composition	Proposed Identification	λ_max_ (nm)
weld (*Reseda luteola* L.)	1	9.6	593	503, 575, 473, 383	C_27_H_30_O_15_	apigenin-*C*-diglucoside	272, 335
	2	9.9	609	447, 285	C_27_H_30_O_16_	luteolin-*O*-diglucoside	268, 336
	3	10.6	609	447, 285	C_27_H_30_O_16_	luteolin-3,7′-*O*-diglucoside	268, 341
	4	11.3	447	285, 284	C_21_H_20_O_11_	luteolin-7-*O*-glucoside	268, 349
	5	12.2	447	285	C_21_H_20_O_11_	luteolin-*O*-glucoside	268, 337
	6	12.2	431	311, 269, 268	C_21_H_20_O_10_	apigenin-7-*O*-glucoside	266, 348
	7	12.4	461	341, 299, 284	C_22_H_22_O_11_	chryoseriol-*O*-glucoside	266, 348
	8	12.8	447	285	C_21_H_20_O_11_	luteolin-4′-*O*-glucoside	268, 342
	9	14.2	285	257, 217, 199, 175, 151	C_15_H_10_O_6_	luteolin	255, 349
	10	15.3	269	225, 151, 117	C_15_H_10_O_5_	apigenin	267, 337
	11	15.5	299	284, 256	C_16_H_12_O_6_	chryoseriol	266, 347
Persian berries (*Rhamni maturi*)	12	9.4	609	447, 285	C_27_H_30_O_16_	kaempferol-*O*-dihexoside	268, 325
	13	11.0	755	609, 463, 301	C_33_H_40_O_20_	quercetin-*O*-dirhamnoside-glucoside	256, 356
	14	11.6	739	593, 447, 285	C_33_H_40_O_19_	kaempferol-*O*-dirhamnoside-glucoside	266, 348
	15	11.9	609	447, 301	C_27_H_30_O_16_	quercetin-*O*-rhamnoside-glucoside	256, 350
	16	12.2	447	301, 211, 151	C_21_H_20_O_11_	quercetin-*O*-rhamnoside	257, 349
	17	13.4	769	623, 447, 315	C_34_H_42_O_20_	rhamnetin-*O*-dirhamnoside-glucoside	257, 357
	18	14.0	301	232, 151, 121	C_15_H_10_O_7_	quercetin	255, 360
	19	14.2	783	637, 491, 329, 314	C_35_H_44_O_20_	rhamnazin-3-*O*-dirhamnoside-glucoside	256, 356
	20	14.5	637	329, 314, 299	C_29_H_34_O_16_	rhamnazin-*O*-rhamnoside-glucoside	250, 355
	21	14.8	623	461, 315	C_28_H_32_O_16_	rhamnetin-3-*O*-rutinoside	257, 347
	22	15.2	285	257, 151	C_15_H_10_O_6_	kaempferol	266, 354
	23	16.7	315	300, 272, 244, 165	C_16_H_12_O_7_	rhamnetin	256, 371
	24	18.0	299	284, 271, 256, 243	C_16_H_12_O_6_	rhamnocitrin	266, 367
	25	18.2	329	314, 301, 299, 286, 271	C_17_H_14_O_7_	rhamnazin	256, 371
	26	20.1	269	241, 225, 197	C_15_H_10_O_5_	emodin	252, 286
Brazilwood	27	8.9	283	271, 255, 229, 211	C_16_H_12_O_5_	brazilein	290, 440
	28	9.3	303	273, 229	C_16_H_16_O_6_	protosappanin B	287, 255
	29	9.3	285	163, 135, 121	C_16_H_14_O_5_	brazilin	-

**Table 3:** Thermal decomposition temperatures of natural lake pigments (T_05_, T_10_, T_20,_ and T_50_) (standard deviations: T_05,10,20,50_ ± 3 °C, char residue ± 2%).

Sample	T_05_ (°C)	T_10_ (°C)	T_20_ (°C)	T_50_ (°C)	Char Residue 600 °C (%)
BW	130	217	265	424	35
PB	142	202	241	334	30
W	118	139	190	316	29
BW/Al	597	-	-	-	95
PB/Al	239	-	-	-	93
W/Al	174	-	-	-	93
W/Sn	196	-	-	-	93

T_05,10,20,50_—decomposition temperatures (5%, 10%, 20%, and 50% of sample mass).

**Table 4:** Digital images and color coordinates of EN films colored with natural lake pigments.

Sample	Image	L	a*	b*
EN	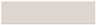	86.52	0.58	5.06
EN/BW/Al	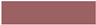	47.52	23.87	9.57
EN/PB/Al	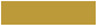	65.72	3.41	53.16
EN/W/Al	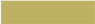	72.27	−6.31	40.96
EN/W/Sn	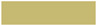	74.85	−7.31	38.09

L—lightness; a*—red-green color coordinate; b*—yellow-blue color coordinate.

## Data Availability

Not applicable.
